# Parents’ experiences with the Circle of Security Parenting (COSP) intervention – a scoping review of qualitative studies

**DOI:** 10.1080/17482631.2026.2644584

**Published:** 2026-03-20

**Authors:** Kirsten Gudbjørg Øen, Veronica Velde Wold, Hanne Cecilie Braarud, Ingvild Sundfør Rasmussen

**Affiliations:** a Department of Public Health, Faculty of Health Sciences, University of Stavanger, Stavanger, Norway; b Aksdal Well Child Clinic, Tysvær Municipality, Aksdal, Norway; c Regional Center for Child and Youth Mental Health and Child Welfare, at NORCE Norwegian Research Centre AS, Bergen, Norway; d Haugesund Well Child Clinic, Haugesund Municipality, Haugesund, Norway

**Keywords:** Circle of Security Parenting, (COSP), scoping review, parents’ experiences, attachment theory

## Abstract

**Background:**

Research on the Circle of Security Parenting (COSP) program is limited.

**Aims:**

The current study was performed to map and synthesize the existing qualitative literature on parents’ experiences of participating in the COSP to address gaps in the current knowledge base.

**Methods:**

A scoping review was carried out. Sixteen qualitative studies with samples from well-baby clinics, community health care, child welfare services, specialist mental health care services for children, and specialist mental health care for adults were included in this review. The sample included 172 informants, mostly women.

**Results:**

Thematic content analysis found three main themes and eight sub-themes. Theme 1: Parents’ journey of self-reflection, self-development, learning and understanding of the parenting role. Sub-theme: (1a) COSP as a source of increased self-understanding and security in the parenting role, (1b) emotionally demanding processes for parents. Theme 2: COSP, a source of changes in the child and parent‒child relationship and interaction with others. Sub-theme: (2a) COSP assessed as having a positive impact on the child. (2b) COSP assessed as improving parent‒child interaction. (2c) COSP as a source of conflict resolution. Theme 3: COSP is assessed as positive, yet sometimes adaptations are needed. Sub-theme: (3a) Parents experience the COSP as useful for their everyday lives and recommend the program to other parents. (3b) Parents have comments and suggestions regarding the implementation of the COSP. (3c) The COSP is recommended by parents outside the target group, but adaptations are required.

**Conclusions:**

The review shows that parents find the COSP intervention to have valuable potential as a health-promoting intervention, but the program should be used with careful consideration to non-target groups.

## Background

The Norwegian Institute of Public Health (2016) estimates that 15–20% of Norwegian children and adolescents experience impaired functioning because of mental health problems (The Norwegian Institute of Public Health, 2016). Parental psychological distress appears to be associated with adverse child mental health outcomes (Amrock & Weitzman, 2014). How parents care for and meet the child’s needs is crucial for physical and mental growth throughout life, and a positive emotional climate between the child and close caregivers is one of the prerequisites for healthy development (Abrahamsen, 2015). The transition to parenthood might be associated with challenges to a greater or lesser extent. Stress and anxiety are experienced in both mothers (McCarthy et al., 2021) and fathers (Baldwin et al., 2018). Parental self‐efficacy has been identified as a key factor that influences parent and child well‐being (Albanese et al., 2019). Strengthening parents’ feelings of competence and mastering in their caregiving should therefore be a key objective. Parents seek inspiration and advice through various channels, and some seek or are recommended professional parental guidance (Bøyum & Stige, 2017).

Attachment is conceptualized as the innate predisposition to develop emotional bonds to persons perceived as stronger and wiser than themselves (Bowlby, 1969). Bowlby proposed that the attachment system developed through evolution to enhance closeness to a caregiver. The availability of a safe haven and the regulation of the child’s distress increase the child’s chances of survival. From a longer perspective, the quality of the child‒caregiver interaction lays the foundation for different outcomes, such as the child’s development of self-regulation and self-identity. Early interactional experiences with caregiver also form the child’s expectation of the caregiver’s availability (Mikulincer et al., 2003).

Mary Ainsworth developed the categorization of infant attachment behaviour patterns into secure, insecure-avoidant and insecure-ambivalent (Ainsworth, 1963). Later, Main and Solomon described a fourth category, disorganized attachment (Main & Solomon, 1986). Although infancy is recognized as the period for the development of attachment, Bowlby suggested that the attachment system is active during the entire lifespan and is seen as a period in which humans search for emotional and social support and closeness to meaningful others (Bowlby, 1988). Research acknowledges the quality of attachment between a child and its primary caregivers as a crucial factor for physical, cognitive, social and psychological development and growth at the group level, possibly throughout life and even across generations (Darling Rasmussen & Storebø, 2021; Darling Rasmussen et al., 2019; Girme et al., 2021; Mikulincer et al., 2003; Spruit et al., 2020; Sutton, 2019).

Parents' belief in their own influence and competence as caregivers is considered important for improving communication and reducing parent‒child conflicts (Bøyum & Stige, 2017). Childcare strategies may be transmitted across generations. Parents who experienced poor quality of care in their own childhood, may have developed problematic inner working models affecting their ability to bond, mentalize and respond sensitively to their children (Van IJzendoorn, 1995).

Parental guidance may help parents better understand and meet their child’s needs. The Norwegian National Infant Healthcare Program for children aged 0–5 years emphasizes that public well baby clinics promote physical, psychological, and social development in infants and toddlers (The Norwerigan Directorate of Health, 2023). Public well baby nurses should have a particular focus on early interaction to promote secure attachment and mastery of the parenting role, to correct negative interactions, and to prevent and avert violence, maltreatment, and neglect. National guidelines for parental guidance programs (The Norwegian Directorate of Health, 2017) state that parents must be provided with knowledge guidance about the child's development and the importance of social interaction and attachment. The guidelines state that universal, primary preventive parenting programs can be offered in groups and/or individually with the aim of promoting children’s health by strengthening parents' mastery of the caregiver role (The Norwegian Directorate of Health, 2017). The Circle of Security intervention is theoretically based on attachment theory and has been found to be the most prevalent parenting program in Norwegian health and social welfare services for children and families (Wesseltoft-Rao et al., 2017).

## The Circle of Security Parenting Program

**Figure 1.**
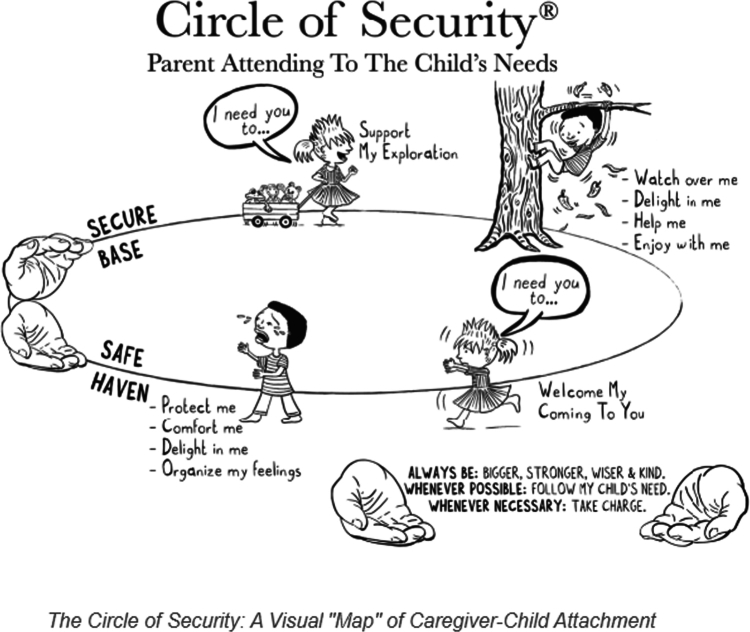
The circle of security for the toddler and preschool years (Circle of Security International, 2022).

The Circle of Security Parenting (COSP) program was designed to offer a more user-friendly and available version of the more intensive therapeutic Circle of Security Intervention (COSI) (Powell et al., 2014). According to the Circle of Security International homepage, the COSP was developed for parents experiencing stress in their caregiving relationships; however, it is described as having a universal appeal and being suitable for all caregivers who want to connect more deeply with their children. It is a manualized attachment-based preventive, psycho-educational program offered to parents in groups or individually, delivered over a minimum of eight weekly 90-minute sessions. Parents are invited to reflect upon pre-made video materials, with COSP facilitators providing reflection questions at determined stops during the video. Central to the program is a graphic model of the Circle of Security (see Figure 1) (Circle of Security International, 2022), picturing the child’s need for nurturing comfort at the bottom of the circle, the child’s need for exploration and autonomy at the top of the circle, and the hands representing the caregiver.

The program seeks to support caregivers’ parental competence and skills by enhancing caregiver mentalization in the parental role. Mentalization can be defined as “the uniquely human ability to interpret the meaning of others’ behaviour by considering their underlying mental states, as well as the capacity to understand the impact of one’s own affects and behaviours on others” (Midgley et al., 2017, p. 15). Parents are also invited to reflect upon how caregiving experiences from their own childhood influence their conscious or unconscious caregiving strategies in relation to their own children. The term “shark music” refers to psychological defense mechanisms that can hinder mentalization by activating the caregiver’s alarm system in response to the child’s harmless expressions of emotional needs (Powell et al., 2014). COSP addresses the balance between parental leadership and boundaries on the one hand, and acknowledges and accepts different feelings with emotional warmth on the other hand (Powell et al., 2014). The program targets how parents can offer successful “repairs” and take responsibility in situations where they have lost the balance of “strong” and “kind” behaviour towards the child. COSP also stresses that the goal is to be a “good enough” parent rather than perfect, and that there are opportunities for growth and development in situations involving mistakes and repair. According to the founders (Circle of Security International, 2025), COSP was designed to enhance the quality of security in relationships between caregivers and children ages 4 months–6 years by building reflective functioning and relational capacity.

They warn that it is not a clinical intervention for severe mental health or trauma issues and should not be offered as a stand-alone intervention in clinical settings, nor when parents are in active crisis, are lacking in basic stability and fundamental needs, and are suffering from severe mental health symptoms or substance abuse issues that might be prevented in reflective work (Circle of Security International, 2025).

The COSP program has been reviewed to have a low cost to set up and deliver (Early Intervention Foundation, 2019). The Early Intervention Foundation in the UK (Early Intervention Foundation, 2019) has rated COSP at evidence level 2+ on a three-point scale, highlighting the need for further research to clarify the program’s potential effects. Similarly, the Norwegian database *Ungsinn* has rated the program at evidence level 2 (theory-based) on a five-point scale, also emphasizing the need for additional research on its effects (Rye & Eng, 2021).

Quantitative research today has relied on small and diverse and often selected samples that yield mixed results regarding the possible effects of the COSP. For example, whereas some studies have found effects on parental mentalization (Maxwell et al., 2021; Shai et al., 2024) or parental reflective functioning (Kohlhoff et al., 2016), other studies have failed to find similar effects (Rasmussen et al., n.d.; Stuart et al., 2025 submitted). Sadowski et al. (2022) found a significant decrease in one of three variables measuring parental reflective functioning for participants who had participated in COSP groups but not for participants who had received COSP individually. Some studies have found decreased self-reported parental stress post-COSP (Cassidy et al., 2017; Kohlhoff et al., 2016; Sadowski et al., 2022), whereas others have not (Shai et al., 2024; Zimmer-Gembeck et al., 2022; Rasmussen et al., n.d.). Some studies have found decreased self-reported parental helplessness (Kohlhoff et al., 2016) or increased parental self-efficacy (Maxwell et al., 2021), while other studies have reported no changes in parental sense of competency (Maupin et al., 2017; Shai et al., 2024). While some studies have found no effect of the COSP on the parent‒child relationship (Maupin et al., 2017), there are reports of changes in parents’ emotions and attitudes towards the child (Kohlhoff et al., 2016; Krishnamoorthy et al., 2020; Maxwell et al., 2021), and fewer unsupportive but not more supportive responses to the child (Cassidy et al., 2017).

The review above indicates that the current evidence of the possible effects of the COSP remains uncertain. Quantitative methods use standardized questions that do not allow informants to present their own subjective context, their interpretation of their experiences and the way they create meaning in their situations with their own words. Qualitative data on the COSP will thus add important details of how services could make adaptions and increase the utility for parents.

Nevertheless, to our knowledge, there is a lack of systematic review of qualitative research on parents’ user experiences with COSP. A scoping review is commonly used to provide a comprehensive summary of a topic, so as topic to identify the key concepts, sources of evidence, and gaps in the research (Peters et al., 2021, p. 4–5). In this study, we wanted to explore parents’ experiences with the COSP with the parents’ voices. Thus, the aim of this study is to map and synthesize the existing qualitative literature on parents’ experiences with the usefulness of participating in the COSP. Throughout the process, we focused on maintaining an open approach to preserve the user perspective when identifying common themes, contextual factors, and other factors that might be important to parents participating in the COSP intervention to enlighten gaps in the current knowledge base.

## Aim

The aim of this study is to map and synthesize the existing qualitative literature on parents’ experiences of participating in the COSP. Throughout the process, we focused on maintaining an open approach to preserve the user perspective when identifying common themes, contextual factors, and other factors that might be important to parents participating in the COSP intervention to enlighten gaps in the current knowledge base.

This study is part of a larger Norwegian public health project, called “Secure Parents at Haugalandet”, which aim to offer the COSP universally to parents of pre-school children. The background for the project was a combined experience of many parents seeking support in the parental role, a high proportion of families with interventions from local social welfare services (Statistics Norway, 2023), along with high levels of mental health challenges among children and adolescents nationally (The Norwegian Institute of Public Health, 2018). To meet these challenges, five municipalities wanted to prioritize early population-oriented intervention with the aim of strengthening protective factors that contribute to good mental health, well-being, and resilience through an intervention aiming to reduce parental stress and promoting deep emotional connections between parents and their children (Haugesund Kommune, 2021).

Research questions:

1. How do parents experience the usefulness of the COSP intervention?
2. Are experiences comparable across studies with different target groups?

## Method

### Design

A scoping review can, compared to a systematic literature study, build upon richer data material to answer the questions as it can choose which studies to include more freely. This is an advantage if there are few published studies on the subject (Arksey & O'Malley, 2005), which is the case for the COSP.

As our aim was to explore parents’ experiences with COSP and let parents’ voices be heard, we only searched for qualitative data. To obtain a broad impression of the parent’s experiences rather than experiences related to a specified topic, the six steps of Arksey & O’Malley’s (2005) framework for scoping review were followed. For further information about the method, see the PRISMA Scoping Review Checklist (Tricco et al., 2018).

In order to have an effective search strategy (see also Levac et al., 2010), our broad research question was combined with a clearly articulated scope of investigation, including definition of the concept, target group and results of interest. To identify relevant studies, the focus was limited to qualitative studies where parents were given open questions or the opportunity to freely share their experiences after participating in the COSP.

The number of databases was limited to a main search in “EBSCO”, with the databases “Academic Search Premier”, “Cinahl” and “Medline”, and a simple search in “PsycINFO”. The flow diagram (Attachment 1) shows the search history. A simple search for master’s theses was performed in Oria, which is considered to be a good search location for identifying relevant master's studies. These keywords were used: “circle of security parenting +parenting experiences”. This yielded 13 hits, and five were included after reading the full text. The last search was performed on August 22^nd^, 2025.

“PICO” (Table I), a tool developed for the purpose of structuring and documenting search processes, was used to form the starting point for keywords and terms. PICO is an acronym targeting four different potential components of a health question used in Cochrane review research (Patient, Intervention, Comparison, Outcome), providing the specific who, what, when, where, and how, of an evidence-based health-care research question (Cochrane Library, 2026).

**Table I.** PICO framework: overview of search words/key words.

Whom or what is the research aeras	Key words
P = Population/problem	Parents, caregiver, mother, father
I = Intervention	COS-P, Circle of Security-Parenting
C = Comparison	Not relevant
O = Outcome	Experience, behaviour, secure, perception, attitude, view, feeling, perspective, mastery, parent child relation

The inclusion and exclusion criteria and decisions of the specific variables to collect were decided through discussion between the 1st and 2nd author.

To increase the quality of the study, one colleague pilot tested our used extraction tools described and ended with the same included studies as we did.

All qualitative studies with open-ended questions about the parents' own experiences written in English, Danish, Swedish and Norwegian were included. Studies investigating other versions of the Circle of Security apart from COSP were excluded. There were limited peer-reviewed publications on the topic; however, we chose to include several highly relevant master’s theses. Note that a scoping study does not seek to assess the quality of evidence and consequently cannot determine whether studies provide robust or generalizable findings (Arksey & O'Malley, 2005).

The analyses were performed by mapping relevant information from primary research reports, and synthesizing and interpreting the data by shifting, mapping, and sorting material according to our central research questions and themes. The present study focused on statements originating from participating parents, in addition to relevant information about the study.

Arksey and O'Malley (2005) recommend the use of both a descriptive numerical analysis and a thematic analysis. The numerical analysis provides an overview of the included studies (see Table II) and participant characteristics (see Table IV).

**Table II.** Inclusion and exclusion criteria.

	Criterion	Grounds
Inclusion criteria	No limitation in relation to the year of publication	The intervention is relatively new, so all publications are seen as relevant
	Qualitative studies that include open-ended questions about the parents' own experiences	Experiences, to which the study relates, are most evident when the participants are given the opportunity to provide a free description
	All studies available in the selected databases	In SCOPING reviews, there is no requirement for peer-reviewed articles
	Research articles in English, Norwegian, Swedish, or Danish	To include nuances in the language, it is important that the author knows the language well
	Parents’ perspectives	The study explores parents' experiences
	Parents who have received the intervention Circle of Security Parenting	There are different COS interventions. This study solely explores experiences after participating in COSP
Exclusion criteria	Studies of other interventions than COSP	Only the COSP intervention is under exploration
	Studies without qualitative methods	Delineate

The qualitative data analysis followed Braun & Clarke's description (Braun & Clarke, 2006) by identifying, analyzing, and reporting patterns (themes) within the data. The themes were sorted in an interactive process between the 1st and 2nd authors following Braun & Clarke’s first 4 phases, with all four authors participating from phase 5.

Phase 1: The data were read repeatedly in an active way, searching for meanings and patterns before coding.

Phase 2: Generating initial codes: The coding was “data-driven” to identify answers to our two research questions. With this inductive approach, we coded all extracts found by marking them with different text colours without using any software tools and kept as much of the context as necessary to secure the right understanding.

### Coding

The qualitative data with the parents’ own voice from each of the included studies were marked with codes by the first and second authors independently. They both verbalized their suggestions for codes. There was no disagreement on the final codes after collaboration and discussions (examples of codes are shown below).

#### Few examples of codes from the different studies

A*. “Some parents were struggling or felt overwhelmed by their child's challenging behaviors”* (15). = Experiences of challenges in the family.

B. *“- and I also think that is because I meet him in a different way than before. I am not as rigid anymore* (8). “So, I've maybe tried to sort of repair more often than before, where I've maybe been like, a little childish, and kind of overlooked her more (13).” = Experiences on changed parenting.

C. “*I basically feel that… as a family, we`re just happier. And I feel closer to my daughter, particularly”* (7). *“- so absolutely the whole family has changed since I took this program”* (16). *=* Experiences of changes in relationships.

D. *“Yes, it is in two ways that children don’t do as you say, children do as you do, and if I am more harmonious and calmer, then the children are too, this thing with punches and kicks and screams has reduced radically since I took this program”* (16). *“We argued a lot before, and we argue very rarely now. And my son has become much calmer. He understands more of what I say”* (8). = Experiences of changes in the child.

E. *“The program has helped me not to go into the authoritarian style, but strengthen me to stay safe in what I think is correct (3) “It’s a parenting course that every pregnant mother and father should do cause it’s just really, it’s really valuable humanizing advice… and it’s a really valuable way of interacting with any human being you come across”* (9). “*I would recommend it to other parents… specifically if I hear or see a parent struggling with the relationship between him or her and her children”* (12)*. =* Parents’ positive experiences.

F. *“The content in the COS-P DVD did not consider parenting children with additional needs”* (2). “*Sometimes I felt like I wasn't good enough as I was, and I lost a little faith in parenting”* (3). “*It’s really hard, cause it’s quite hard in everyday life looking at typically developing children and then seeing that your child’s not and then sitting down for whole course watching it”* (9). *=* Parents’ negative experiences.

G. *“I think the videos, the whole, you know, what you’re watching is not, it’s not dealing with the situation that you’re in”* (9). = Suggestions for changes in the program.

H. *“Sometimes some parts of the content would need a repetition so that all the pieces would fall into place. I would like to go all over again”* (10). = Need for more follow up.

I. “*It was positive that my supervisor, she has a calm personality, she speaks to you with respect. I gained trust in her... she reached out to me in a way that no one has done before. She wants me well, I can see that”* (8). = COSP facilitator is important.

Phase 3: Searching for themes: We re-focused the analysis to a broader level of themes, sorted the different codes into potential themes, and collated all the relevant coded data extracts within the identified themes.

The data were sorted into 4 tables. Each table is sorted into several categories. The first table mapped participants’ motivations for participation in the program and challenges reported by parents’ or families. Table II mapped how the program impacts parents and whether and how parents experienced changes in their child. The third table mapped parents’ experiences of changes in relationships within the family and with others. The fourth table mapped parents’ satisfaction with the program, positive and negative experiences, suggestions for program changes, and differences between different target groups.

Phase 4: Reviewing themes: The themes were narrowed so that the data within themes should cohere together meaningfully with clear and identifiable distinctions between themes. We drew a “thematic map” before re-reading the dataset to ensure that the themes covered the dataset, and that no data were overlooked at earlier coding stages.

During discussions between the authors, an initial thematic map consisting of two main themes and seven subthemes was drawn: (1) The parents have positive experiences with the COSP intervention and would recommend it to other parents, with subthemes: (1a) Parents experience a better understanding of themselves and have gained increased security in their parental role, (1b) Parents experience that the COSP has a positive impact on their child, (1c) Parents experience a positive impact on the interaction with the child, and their communication and relationship with others, and (1d) Parents value the content and working methods of the COSP. 2: Parents have both positive and negative experiences with the implementation of the COSP and have input for improvements, with sub- themes, (2a) Parents experience that participating in COSP create difficult feelings. (2b) Parents have negative experiences with the content and implementation of COSP, (2c) Parents experience the COSP program are useful in their everyday life, but some suggested improvements for the implementation.

Phase 5: Defining and naming themes: We interpreted what exactly “story” each theme told us. We identified two main themes and seven sub-themes and provided analytic names considered concise enough to give the reader a sense of the content of each theme.

Further discussions of the data material led to the conclusion that our initial thematic map did not fit our data material completely, and we returned to further review and refine the coding. After thorough reflections about the various themes and ensuring that we captured the data content that should be included, we identified the essence of interest in each theme. During this process, we strove to stick to the parents’ experiences but interpreted the data at a deeper level, digging deeper into the meaning of the parents’ story. This cooperating process resulted in a final thematic map with new and deeper interpretations of the themes.

Phase 6: Producing the report: We presented the analytic names and emphasized that our description and interpretation of the data enlightened our research questions.

Sort, summarize and report the results:

Information about the various studies, such as country of origin, year, title, and characteristics of the target group, was presented in tabular form in a numerical summary (see Table II and Table IV).

The results of the qualitative thematic analysis (Braun & Clarke, 2006) with the interpreted themes and sub-theme answering the research question are presented by a narrative story adding the studies applicable to the findings in the various sub-themes.

### Authors reflexivity statement

The first author is a public health nurse and researcher on psychosocial work with children and parents. She had no experience regarding the practical use of the COSP; however, her clinical experience involved counselling parents about various themes in various settings. She was the supervisor of the master’s thesis on which this manuscript is based. The second author is a public health nurse working at a child health clinic for children aged 0–5 years. This article is based on her master’s thesis from the public health nursing program (2021/22), written prior to gaining practical experience with COSP. She has since become a certified COSP facilitator and now applies the program in her professional practice. The third author is not trained as a COSP facilitator but is a clinical psychologist and researcher with a focus on developmental psychology and early parent‒child interaction. The fourth author is a clinical psychologist and a certified COSP facilitator. She has experience in clinical practice in municipal mental health services for children and adolescents and was the project manager of Secure Parents at Haugalandet.

The authors reflected and comprehensive discussions on codes and groupings of themes took place throughout the entire research process and contributes to strengthen the validity of the study. Reflexive and more interpretive discussions throughout the process helped us capture themes of interest from the parents’ testimonies. To conclude the discussion and decide on the final themes, the question “what is interesting in what we have found?” became central. The deeper and more interpretive discussions throughout the process helped us to capture themes of interest from the parents’ testimonies.

## Results

The findings are presented in a numerical summary and a thematic summary.

### Numerical summary

In Table III, the selected studies are presented with a brief explanation of the population characteristics. In the remainder of this paper, reference will only be made to the study number, as presented in this table.

**Table III.** Overview of selected studies with characteristics of the study populations.

Study number	(Author, Year) country of origin	Study title	Number of informants	Child age	Characteristics of the participants	Program delivered by
1	Alstergren (2017) Norway	Parenting experiences after COS-*P* (Circle of Security—Parenting)	*N *= 8	0–18	Normal challenges: Typical conflicts, stress in everyday life, single parent, children showing anger	Primary health care
2	Birdsey et al. (2023) England	Piloting the Circle of Security Parenting group with parents of children with a learning disability: An exploratory case study	*N* = 4	5–12	Children with developmental disorders, parenting stress, difficult understanding and meeting the children’s needs	Specialist service, children with learning disabilities team
3	Dingstad (2019) Norway	Circle of Security Parenting—an American program in a Norwegian context	*N* = 8	0–5	Typical challenges, stress due to busy everyday life. Two participants had very difficult relationship with their child, and experiences of inadequacy and worries about own anger	Well-baby clinic
4	Gundersen (2018) Norway	Circle of Security—Parenting. How do parents experience the benefits and relevance of the COS *P* program? A qualitative study	*N* = 4	7–9	Parents with stressful everyday-life and feelings of inadequacy	Specialist service. Children who strive with concentration and hyperactivity, feelings and behaviour, and anger
5	Holte (2018) Norway	Experiences with Circle of Security Parenting in child protection centers	*N* = 7	0–5	Major difficulties with parent-child interaction, and parent’s own mental health and childhood trauma	Family based centers in child welfare services
6	Kimmel et al. (2017) USA	Maternal Experiences in a Parenting Group Delivered in an Urban General Pediatric Clinic	*N* = 12	3–36 months	Mothers with low socio-economic status, psychological problems, and about 40% with experiences of own childhood trauma	Urban general pediatric clinic
7	Maxwell et al. (2021) Australia	Parent and practitioner perspectives on Circle of Security Parenting (COS-*P*): A Qualitative study	*N* = 14	4 months–6 years	Varied combination of participants. Parental stress, handling child behaviour, conflict with wife’s ex-husband, post-partum depression, difficult childhood experiences.	Service not described
8	Michelet and Klevan (2020) Norway	Increased parental coping and tranquility in everyday life A qualitative study on parents’ experiences with participation in the COS-P program	*N* = 4	4–10 years	Characteristics of the participants struggles and experiences not described.	Social child welfare service and mental health team
9	Muddle et al. (2022) Great Britain	Talking with parents of children with learning disabilities: Parents ideas about the circle of Security Parenting program	*N* = 6	5–14 years	Children with moderate to severe learning disabilities, parents trying to understand and be sensitive to their children’s needs.	Specialized mental health service
10	Neander and Risholm Mothander (2015) Sweden	Circle of Security and reflexive parenting: COS-*P* in Sweden—implementation a new psychoeducative parenting program.	Survey (*N* = 28) Interviews (*N* = 9)	3–58 months	Families at risk. Parents suffering from anxiety or depression, got help and support for parenting and child development. High on parenting stress.	Specialized mental health service/family centre
11	Pazzagli et al. (2014) Italy	The Circle of Security Parenting and parental conflict: a single case study	*N* = 1	5 years	Disagreement around parenting, high conflict with the child’s mother. High levels of stress and feelings of not being good enough, the feeling of being rejected by the child.	Private psychotherapist
12	Rose et al. (2018) South Africa	Circle of Security parenting program efficacy for improving parental self-efficacy in a South African setting: Preliminary evidence	*N* = 8	Preschool ages	Participants described that they were not prepared to be parents, and the role came sudden. The role as parent is overwhelming, and they didn’t know how to act.	Not described. Intensive COSP program.
13	Sæther and Glavin (2021) Norway	Engaged parenting”—parents’ experience with the parent training program Circle of Security Parenting, COS-P	*N* = 9	2, 5–12 years	Hyperactive children (2), angry children (3) or withdrawn children (1), challenging cooperation between parents (1), no difficulties (2).	Well-baby clinics
14	Helle et al. (2023) Norway	From seeing difficult behaviour to recognizing legitimate needs—A qualitative study of mothers' experiences of participating in a Circle of Security Parenting program in a public mental health setting.	*N* = 12 women women	0–15 years	Primary diagnosis was mood disorder(*n *= 2), anxiety disorder (*n *= 8), personality disorder (*n *= 1), eating disorder (*n *= 1)	A district psychiatric health center within Public mental health care
15	Kohlhoff et al. (2025) Australia	Circle of Security-Parenting and Parent-Child Interaction Therapy-Toddler: A Qualitative Exploration of Parents' Perspectives.	*N* = 19 mothers	14–24 months	Parents had concerns about their child’s behaviour and/or had difficulty managing their child’s behaviour and were referred by their primary health care provider.	A community-based child behaviour treatment clinic in Sydney
16	Savela et al. (2025) Sweden	Parental empowerment and child–parent attachment: A qualitative study of the Circle Of Security-Parenting Programme.	*N* = 19	0–17 years	No question was asked regarding whether the parents had parental challenges prior to attending the Cos-P.	Cos-P from a health-promotive perspective offered by the Social Services or the Church of Sweden

Table IV shows a summarized overview of population characteristics in the various studies. Studies are divided into three main groups based on description of the context in which the COSP was delivered and subsequently organized into eight subgroups reflecting specific challenges or risks identified in the study populations. The identified challenges or risks were worries for the child because of parents’ mental health problems, child problems, stress and family conflicts, or parents striving in the parental role.

Tables II and IV show that our results on parents’ experiences of participating in COSP rely on data from a wide range of contexts. The 172 informants, mostly women, have participated in COSP program delivered in the scope from primary health care services to mental health care clinics for parents or children, as well as social welfare services.

**Table IV.** Review of population characteristics.

Received COSP via specialist healthcare services			Received COS *P* via primary healthcare services			No information about context for receiving COSP	
2, 4, 5, 8, 9, 10, 11, 14 15			1, 3, 6, 13, 16			7, 12	
Social child welfare. Worries of the child because of parents’ mental health problems, or problems in their parenting role	Child problems	Family conflicts	Parenting stress, busy everyday-life, conflict in the family	Child behaviour problems	Severe problems related to parents’ mental health or challenges in the family system.	Striving as a parent	Parents mental health or challenges in the family system
5, 8, 10, 14	2, 4, 9, 15	11	1, 3, 13, 16	1,13	6, (3)	7, 12	7

### Thematic summary

In the final thematic analysis, three main themes and eight subthemes emerged.

Theme 1: Parents’ journey of self-reflection, self-development, learning and understanding of the parenting role.

Sub-theme:

(1a) COSP as a source of increased self-understanding and security in the parenting role.
(1b) *Emotionally demanding processes for parents.*

Theme 2: COSP as a source of changes in the child and the parent-child relationship and interaction with others.

Sub-theme:

(2a) COSP assessed as having a positive impact on the child.
(2b) *COSP assessed as improving parent-child interaction.*
(2c) COSP as a source of conflict resolution and interaction with others.

Theme 3: COSP is assessed as positive, yet sometimes adaptations are needed.

Sub-theme:

(3a) Parents experience COSP as useful to their everyday lives and would recommend the program to other parents.
(3b) *Parents have comments and suggestions regarding the implementation of COSP.*
(3c) COSP is recommended by parents outside the target group, but adaptations are required.

Theme 1: Parents’ journey of self-reflection, self-development, learning and understanding of the parenting role.

(1a) COSP as a source of increased self-understanding and security in the parenting role.

Several studies contained statements from the parents that they felt more secure in the parenting role, largely explained by acquired new knowledge that made them more confident in how to handle various situations (1, 2, 3, 4, 5, 6, 7, 8, 10, 11, 13, 14, 15, 16). One study revealed divided opinions among participants, with some reporting increased self-confidence in their parenting role, while others did not have the same experience (4). Many parents expressed experiencing less stress in the parenting role (3, 5, 8, 10, 11, 16).

Parents also reported that the intervention increased their awareness and provided a new understanding of their children's needs. These new skills made it easier for them to see the connection between the children's needs and their own behaviour (1, 3, 4, 5, 6, 7, 8, 10, 11, 13). The parents experienced that they had become more aware of their children's different expressions of emotion and how to support both difficult and positive children. New insights and knowledge meant that parents had become more aware of their children's need for exploration, and how the parents themselves could be an obstacle to the child’s exploratory behaviours. They now did more to support their children in their exploration (1, 3, 4, 5, 10, 13, 16).

Some parents described an increased awareness and understanding of their own experiences and emotions, reactions, behaviours and needs (14). Parents had acquired a new understanding of how their own experiences affected them personally and/or in the parental role (1, 3, 4, 5, 6, 7, 8, 10, 11, 13, 14). With these new insights, parents experienced that they were more aware of their own emotions and reactions and had gained new tools to manage their own emotions or temperaments (1, 3, 4, 5, 6, 7, 8, 9, 10, 13, 14). Parents appreciate the opportunity to reflect on their own behaviour individually and with others (16).

(1b) Emotionally demanding processes for parents

Several parents reported that becoming aware of mistakes they had made in the past and the significance this could have had for the child resulted in guilty feelings (3, 4, 5). Some parents expressed that COSP had made them feel that they were not good enough at parenting (2, 3, 4).

Parents participating in COSP through specialized mental health care for adults stated that COSP had provided increased understanding and acceptance of their own developmental history and expressed a wish to do things differently than their parents, and to disrupt the problematic emotional patterns that had been repeating across generations (14). Some parents found it difficult to reflect on their own childhood and how these experiences affected them as parents. They found it painful to look back, difficult to reflect on mistakes their own parents had made, or difficult to experience that their childhood negatively affected their parental role (1, 5, 7, 10, 8, 14). Attending the COSP as a couple and listening to their partner’s story was extremely difficult for some because they had to support their partner and simultaneously have their child in focus while dealing with their own emotions (7).

The focus and reflection upon painful topics were experienced as shocking, upsetting, or confrontational by some parents (4, 5, 7, 8, 10). Other parents expressed that they considered carefully what personal information they wanted to disclose in the group (1). Instead, they reflected upon what the other parents shared in the group, but also their own experiences or reflections with their partner between group sessions.

According to this sub-theme, the facilitator plays an important role in mediating the parents' experience. Although, as described in theme 3a, most parents felt supported by the facilitators, some parents had negative experiences where they did not perceive the contact with the facilitator or the group as positive (3, 4, 5, 7, 9). Additionally, parents’ initial motivation for participation affects their expressed utility of the program (4, 5).

Some parents experienced that the COSP contributed to an increased level of conflict between themselves and their partner. Both parents’ participation has been noted as beneficial (1, 13). Some who participate alone find it difficult to convey knowledge to non-participating parents without appearing to be criticizing (1, 10). The participating parents experienced that their own will to change conflicted with the non-participating parents’ resistance to change and that, as a result, the non-participating parent became angrier after the intervention (13). In one case where disagreements about upbringing are described as a source of conflict in the parents’ relationship, the absence of change was perceived as provoking increasing critique from the other parent after both parents participated in the COSP intervention (3).

Theme 2: COSP as a source of changes in the child, the parent‒child relationship and interaction with others.

(2a) COSP experienced as having a positive impact on the child

Only a few studies explored COSP’s influence on the participants’ children, and they also varied in their descriptive details. However, in some studies, parents reported experiencing their child/children as more satisfied or happy after their participation in the COSP (1, 8, 10). Parents also perceived the children to be more secure as they expressed more emotions (7, 8 10, 13). Several studies have reported that parents face challenges related to conflicts and child behaviour before they participate in the COSP. Some of these studies reported that parents encountered fewer behavioural challenges, and/or that incipient conflicts were resolved at an earlier stage, consequently resulting in a reduction in outbursts of anger post intervention (1, 4, 7, 8, 10, 11, 13). Findings from individual studies reported that parents perceived that their children gained better self-esteem (10), better trust in their parents (8), became more independent (10), gained better social skills (11) or showed improved behaviour and emotion regulation skills (15). Some studies also stated that parents experienced positive changes in their children without further elaborations (4, 5, 13). One study reported that parents perceived the reason behind why they did not experience any change in the child’s reaction was that the child/children had certain expectations of the parents’ reactions and needed time to become accustomed to the parent’s new interactive behaviour (3). Parents from a different study also reported that while they still experienced continued challenging behaviour in their children, their own reflections on the behaviours had changed (15).

(2b) COSP experienced as improving interaction with the child

Parents experienced a positive impact of the COSP on their interaction with the child. Several of the studies reported that parents felt that they had gained a closer relationship with their child or that the parent‒child relationship had strengthened (1, 4, 7, 10, 11, 13, 15). One of these studies also revealed that a few parents did not experience any positive influence on the parent‒child relationship (4). The parents expressed that they spent more time and enjoyed being with the child or felt more emotionally available when interacting with the child (1, 3, 4, 5, 7, 8, 9, 10, 11, 13). Some of these studies found that parents experienced fewer conflicts and calmer everyday lives (1, 8, 10, 13). In three of the studies, parents expressed that they had become more conscious of “making repairs” after making “failures” in the parenting role (5, 10, 11).

The parents reported that they had changed the way they met their children in difficult situations, described as the effect of “time in” instead of “time out”. They had increased their understanding of the use of closeness and support as opposed to punishment/sanctions when the children displayed challenging behaviour. Apparently, parents took to heart and utilized the term “bigger, stronger, wiser and kind” in everyday life (1, 3, 4, 5, 7, 8, 9, 10, 11, 13).

(2c) COSP as a source of conflict resolution and interaction with others

Parents also expressed a positive impact of COSP on their communication and relationship with other people. Parents stated that COSP offered guidance and an understanding of managing conflicts and setting boundaries (16). In several studies, participants perceived that participation in the COSP program influenced their relationship with their partner, colleagues, and other adults. This mainly concerned the ability to understand other people, awareness of how they communicate, or that they became more confident in talking about topics that were previously difficult to talk about (1, 3, 4, 5, 6, 7, 9, 11, 16). The participants also stated that COSP resulted in a common language between parents (1, 7), better cooperation (4, 7, 11), and better relationships with their partner or people in a work context (7). Participating in COSP as co-parents was perceived as rewarding because the intervention stimulated discussions of relevant topics (10).

Theme 3: COSP is assessed as positive, yet sometimes adaptations are needed.

(3a) Parents experience COSP as useful to their everyday lives and would recommend the program to other parents

Statements from the parents showed that the program content was understandable and meaningful (1, 3, 5, 7, 10, 12, 13). The use of graphics and key concepts seemed to make it easier for parents to visualize, understand and remember the material and apply the theory in everyday life (1, 3, 4, 5, 7, 10, 11, 13). Different opinions about some concepts are further elaborated below.

The combination of theory, video and reflection was identified as effective and highlighted as contributing factors to parents’ satisfaction. The videos and reflections made it easier to relate to and remember the material (1, 3, 5, 7, 12), and the reflections between the parents/group and the providers seemed to be particularly important for a positive experience for the parents. In most of the studies, parents discussed the significant role of the facilitator. Parents spoke positively about the facilitator and acknowledged their competence (15). When reflecting on the parental role, the parents experienced feedback and support, which contributed to increased insight and understanding around everyday challenges and the parenting role (1, 2, 3, 5, 6, 7, 8, 9, 10, 13). Parents valued the support and encouragement they received from facilitators and other parents in the group (1, 2, 3, 5, 6, 7, 8, 9, 10, 13, 15). It appears to be important to have a good relationship with the facilitator, and it was appreciated when the facilitator shared his/her own experiences, without appearing to be an expert but rather as “one of them” (3, 5, 7, 8, 13). In some studies, parents express the importance of clearly communicating that there is room for error and that emphasis is placed on the importance of being “good enough” (3, 5, 7, 8).

In several of the studies, the parents expressed that they use new knowledge in everyday life, and several said that they see the circle in their mind all the time. The perceived usefulness was generally positive in most of the studies, even though some also tended to have critical comments on parts of the program (1, 5, 6, 7, 8, 10, 11, 13).

Generally, there was little variation in the opinions about the intervention within the various studies. Altogether, parents would recommend the program to other parents, with some even stating that it should be compulsory (1, 3, 5, 8, 9, 10, 12). None of the participants stated that they would not recommend the COSP intervention to other parents. Some parents expressed that it could be difficult to use new knowledge in everyday life, depending on their patience and emotions from day to day, with a busy everyday life making it easy to fall into “old habits” (1, 2, 3, 5, 7, 10, 13).

(3b) Parents have comments and suggestions for the implementation of the COSP

In three of the studies, parents experienced that they benefited more from the part of the program that focused on the parent and the parenting role than the first three chapters that focused on the child (3, 10, 13). Other parents find parts of the content, and in particular the concept of “shark music”, that are difficult to understand (3, 7, 10).

Timewise, completing the COSP intervention for parents can be demanding. Although this is not particularly highlighted in the selected studies, the views expressed in two of the studies indicate satisfaction with individual adaptations (1, 3). Some parents described time commitment and distance to the treatment facility as treatment barriers (15), yet there was a recurring desire for follow-up sessions or further contact with the group/facilitators post intervention (1, 6, 7, 8, 10, 13).

(3c) COSP is recommended by parents outside the target group, but adaptations are required

Although most parents from non-target samples appreciate the COSP, they demand adjustments. The experiences from participation and benefits from COSP varied among participants from specialist mental health services (4, 5, 9, 10), and although the videos used in the program are valued and described by many participants in general as important for the understanding and benefit of the program, several parents of children with identified difficulties and neurodevelopmental disorders stated that they found it difficult to relate to the content, and wished for video examples that they could better relate to (2, 5, 9, 10).

In two studies with parents of children with neurodevelopmental disorders, parents described how the intervention contributed to increased grief and difficult feelings of having a “different child” (2, 9). They found it difficult to reflect on videos and examples showing children with very different needs and challenges than those of their own child. This led to increased grief over not having the life they could have had and the limitations they and their children experienced. One parent referred to the circle of security as “the circle of sadness” (2). These parents problematized the COSP mantra “always be bigger, stronger, wiser and kind” and the concept of “shark music”, as they experienced that their children made them feel constant alert and demanded them to be one step ahead to forefront dangers their children had no concept of (2, 9). Nevertheless, the same parents stated that they found the COSP useful and would recommend it to other parents but underlined a need for being better informed about the intervention before attending and more support during the intervention (9) and suggested changes to the program so that it is more adapted to their children’s needs (2, 9). Also, some parents receiving COSP through child protection services expressed a need for more support during the intervention (5). In some studies, there was varying feedback about the usefulness of the intervention afterwards (4, 9). While one parent receiving COSP through specialist health care for children thought the program was too “basic” (4), a different study from a similar sample revealed a desire for more time to go through difficult topics, with one parent expressing a wish to repeat the whole program (10).

## Discussion

The purpose of this scoping review was to map and synthesize the existing qualitative literature on parents’ experiences of participating in the COSP, with a focus on identifying common themes, contextual factors, and gaps in the knowledge base. The findings suggest that the COSP eight-week intervention appears to be beneficial for most parents, and that parents generally have very positive experiences with participating in the COSP intervention. Parents across studies stated that they would recommend COSP to other parents, with some even suggesting that it should be mandatory for all parents (1, 3, 5, 8, 9, 10, 12). Even though some previous quantitative studies found no reduction in parental stress after COSP (Shai et al., 2024, Zimmer-Gembeck et al., 2022; Rasmussen et al. n.d.), our findings support contrasting findings from other studies (Cassidy et al., 2017; Kohlhoff et al., 2016; Sadowski et al., 2022), as many parents expressed an experience of reduced stress in the parenting role after participating in COSP (3, 5, 8, 10, 11).

Additional positive gains expressed by the parents included increased awareness and the experience of a new understanding of their child’s needs (1, 3, 4, 5, 6, 7, 8, 10, 11, 13, 14), increased awareness and new tools to manage their own emotions or temperament (1, 3, 4, 5, 6, 7, 8, 9, 10, 13, 14), and the experience of increased confidence in how to handle various situations (1, 2, 3, 4, 5, 6, 7, 8, 10, 11, 13, 14). The study findings are comparable with those of Maxwell, McMahon and colleagues (2021), who reported a reduction in parental helplessness post intervention. Some parents expressed the experience of being more confident and competent as caregivers (8, 14, 15, 16). Parent self-efficacy has been found to be a key factor impacting parent and child well‐being, the parent–child relationship, parental mental health, and child development (Albanese et al., 2019), which also are aims of COSP (Circle of Security International, 2022).

Our study revealed that many parents experienced that they had gained a new understanding of themselves and how their own experiences affected them personally and/or in their parental role (1, 3, 4, 5, 6, 7, 8, 10, 11, 13, 14). Such insight has been found to help parents in their parental role in the more intensive Nurse Family Partnership program, resulting in improvements in parents’ and infants’ lives (Miller, 2015). The parents in our study also reported an increased ability to understand other people, increased awareness of how they communicate, or that they had become more confident in talking about topics that were previously difficult to talk about (1, 3, 4, 5, 6, 7, 9, 11). Together, the findings suggest that the intervention may have affected parents’ mentalizing abilities and their ability to understand, cope with and share emotions, and lend support to previous studies, which have demonstrated that the COSP intervention led to increased embodied mentalizing abilities in a small sample of low-risk mothers (Shai et al., 2024), significant increases in parental reflective functioning and behavioural and emotional responsiveness, and increased parental mentalizing (Maxwell et al., 2021; Shai et al., 2024) and self-efficacy regarding empathy and affection toward the child (Maxwell et al., 2021).

In our study, parents reported specific (1, 7, 8, 10, 11, 13) or unspecified (4, 5, 13) positive child behavioural and emotional changes post intervention, and decreased child behavioural challenges and outbursts of anger (1, 4, 7, 8, 10, 11, 13). It is unknown whether it is the parents’ view of the children that changed or the children themselves. Randomized studies have revealed reductions in a related construct: parents’ negative feelings (rejection and anger) toward the child (Maupin et al., 2017) and reduced hostility toward the child (Maxwell et al., 2021). One possible explanation could be that new ways of interacting with the children due to a different parental perspective resulted in changes in the children’s psychological wellbeing and/or social interaction strategies. However, caution must be taken, as the results are based on small samples of parental self-reports. Observations of children’s behaviour should be explored in future research.

In contrast to quantitative studies, which have found no effect of COSP on the parent‒child relationship (Maupin et al., 2017), our review revealed that the parents themselves experienced that they had changed their behaviour towards the child. Many parents reported that the parent‒child relationship had strengthened (1, 4, 7, 10, 11, 13, 15) and reported spending more quality time with their child and being more emotionally available when interacting with the child (1, 3, 4, 5, 7, 8, 9, 10, 11, 13). The parents also reported that they had changed the way they met their children in difficult situations due to increased understanding of the child’s need for closeness and support instead of punishment/sanctions when the children displayed challenging behaviour. Thus, the findings of the present review indicate that parents had taken to heart the COSP mantra of being “bigger, stronger, wiser and kind” and that they used it in their everyday life (1, 3, 4, 5, 7, 8, 9, 10, 11, 13) and support results from quantitative studies (Kohlhoff et al., 2016; Krishnamoorthy et al., 2020; Maxwell et al., 2021) that the COSP led to changes in parents’ emotions and attitudes towards the child, but stands in contrast to the study by Cassidy et al. (2017), which reported fewer unsupportive but not more supportive parental responses to the child. Several parents reported increased awareness of their children's need for exploration, which in turn led to more parental support for the child’s exploratory behaviour (1, 3, 4, 5, 10, 13). More research on this issue could highlight whether the COSP intervention could be an effective program to prevent anxious behaviour in children (Emerson et al., 2019).

Our findings suggest that even though the COSP is considered affordable (Early Intervention Foundation, 2019) and user-friendly (Powell et al., 2014) parental intervention suitable for low-risk families, parents are invited to undergo complex processes of examining their own behaviour as well as their own upbringing and to question implicit cultural values, which can be emotionally demanding. The findings suggest that the part of the program that concerns such topics is considered particularly beneficial (3, 10, 13) but also complex and sometimes difficult to fully grasp (3, 7, 10). Our study emphasized that parents from the majority of the studies highlighted the importance of the role of the facilitator in creating a safe, nurturing and group environment (1, 2, 3, 4, 5, 6, 7, 8, 9, 10, 13, 14, 15, 16), ensuring that all participants obtain the right understanding of the material (2, 3, 7, 9, 10), and handling participants’ possible feelings of sorrow (1, 5, 7, 8, 9, 10) and guilt (2, 3, 4, 5) with careful consideration. Parents value how facilitators are humble and share their own experiences to minimize professional gaps (3, 5, 7, 8, 13) and emphasize the goal of “good enough” parenting (3, 5, 7, 8), perhaps by skillfully role modelling this topic. We have identified the following three barriers for successful implementation in previous studies: 1) insufficient workplace accommodation, 2) insufficient access to quality supervision at the workplace (Maxwell et al., 2021), and 3) a concern among unexperienced COSP facilitators that the program will be burdensome for parents (Cooper & Coyne, 2020). We would recommend that future studies further examine how previous experience and/or professional supervision may influence successful implementation and participants’ experiences with COSP.

While results from the study by Sadowski et al. (2022) suggest favourable effects of group participation as opposed to receiving COSP individually, and informants in our review in general valued the support of the group, our study also revealed that some parents expressed a need for the program to be delivered individually, at home, or with the possibility to bring infants (1, 3). Note that even though it can be demanding for parents of small children to participate in an eight-week intervention, parents from most of the studies expressed a wish for more, as opposed to fewer, sessions (1, 6, 7, 8, 10, 13). This finding must be considered with respect to the cost‒benefit intention of the COSP as a low-cost intervention. Future research should not only include robust effect studies of the COSP as a public health intervention but also investigate the optimal number of sessions required for the intervention to be effective. Are follow-up sessions as requested by participants a “need to have”, or “nice to have”?

The findings also show that some parents had a strong recommendation of COSP and stated that all caregivers involved in the daily care of a child should be encouraged to participate in the COSP intervention to promote mutual understanding and parental cooperation (1, 10, 13). COSP facilitators should emphasize how parents can support each other in the parenting role and offer guidance when informed about or sensing parental conflicts (3). The possible effects of the COSP intervention on the relationship between parents had little focus in the included studies; however, statements from parents suggest that it led to both increased cooperation and mutual understanding (1, 13), as well as increased conflicts (3). It would be interesting to conduct more research on this topic in future studies.

This scoping review also acknowledges a careful consideration about whether and how to administer the intervention to caretakers outside of the target group. According to Circle of Security International, COSP was not designed for families at risk (Circle of Security International, 2022). Well-trained and supported COSP facilitators can engage at-risk families using the COSP mainly to motivate them to perform more targeted interventions or as a support to other ongoing therapeutic interventions (Circle of Security International, 2022). Two studies in our review targeted parents of children with developmental disabilities (2, 9). These parents experienced difficult feelings in relation to participating in the program and expressed experiences of grief over having a “different child”. Despite this, they still recommend participation in COSP to other parents and made suggestions for adaptations to better suit their situation (2, 9). A recent systematic review of the efficacy of various versions of the COS intervention for parents of children with developmental delay or intellectual disability (IDD) (McBride et al., 2025) revealed that only four studies were included, yielding very different results. The authors concluded that “while COS concepts and the space for reflection that COS provide can be beneficial for parents, COS in its current format also highlights the differences and additional challenges in parenting a child with IDD, which can be emotionally challenging for parents” (McBride et al., 2025, p.11). These findings are in line with our findings, highlighting the need to carefully consider how and when to use COSP with parents of children with developmental disabilities. On the one hand, these parents should not be discriminated against by not being offered an intervention that could possibly increase their self-efficacy, as found in two of the studies by Mc Bride et al. (2025); however, it appears that more tailored versions such as the COSI or COSP with adjusted video material could be more beneficial and prevent additional grief of having a different child.

The present study found that while one parent who received COSP through specialist mental health care for children was offended by being offered the program and stated that it made her feel like they saw her as an incompetent parent, and that the program was too basic and not useful to her, other parents who participated in the context of specialist mental health services valued and recommended COSP to other parents in their situation (4, 10). Parents in adult specialist mental health care suggested that COSP as an adjunct to psychotherapy can successfully address vital reflections on parenting and the impact of previous relational experiences for individuals suffering from psychological mental health problems (14). In one study with few informants receiving services from child welfare and psychiatry services (8), the importance of a caring, engaged, calm and safe facilitator who spoke clearly and with respect was highlighted to enhance their ability to reflect. According to COSP, building a therapeutic alliance with families is an important first step (Circle of Security International, 2022). The therapeutic alliance is described as an agreement on therapeutic goals, a consensus on treatment tasks, and a relationship bond between the patient and the therapist (Bordin, 1979).

COSP was not developed as a stand-alone intervention for high-risk populations, although it can provide foundational learning to enhance the effectiveness when combined with other clinical interventions. The Circle of Security International has defined the target group for the COSP as “… parents experiencing attachment-related stress who are motivated to participate”. They argue that “Studies should exclude families in active crisis or those requiring more intensive interventions, as these factors could obscure COSP's specific contributions”. A careful consideration of for whom, why and how the program could have a positive impact should be clearly stated and evaluated in future studies. In addition, although the COSP may appear to be a lightweight manualized psychoeducational intervention suitable particularly for low-risk families, several studies in our review suggest that it may initiate complex emotional processes for its participants, reflecting on their own childhood experiences and vulnerable aspects of their parental role. Additionally, although there is a pre-made video material, a highly important element is participants’ own reflections with their COSP facilitator and/or group of other parents. Such complex processes require both personal and professional skills and experience. We therefore request that all providers of COSP offer facilitators adequate guidance, support and time for the task. We support the conclusions of Mc Bride et al. (2025) that further research is needed to more robustly examine the benefits and limitations of utilizing different versions of the COS with this population. In the meantime, we strongly encourage administrators to carefully consider the total situation of the caregivers and the child, and to properly inform and consider possible necessary adjustments before offering the COSP intervention to parents of children with developmental disorders.

### Strengths and limitations

To our knowledge, this scoping review is the first to intentionally explore experiences of COSP in a diverse caregiver population, which adds valuable knowledge to the evidence base. Using prior research findings might increase the risk for bias. Several steps were taken to mitigate bias and strengthen the validity of our findings. Robust methodologies were followed, including the use of the Prisma-ScR framework, a structured framework for scoping studies, and qualitative content analysis. Two researchers searched for data with the same search strategy and coded the data using the parents’ own statements (rather than the primary researcher’s results) from interdisciplinary research carried out in different professional areas. The data were reported with transparency of the coding and the content analysis, and decisions made during the research process should make reproducibility of the review possible.

In the scoping study procedure, it is not necessary to make a critical assessment of the studies (Arksey & O'Malley, 2005), which offers more freedom in the choice of studies to include. It would be an advantage to assess the quality of the studies included. On the other hand, the included studies had met ethical and quality requirements because they were published.

One limitation is that searches were limited to five databases. More information might have emerged by including several other keywords, other search bases and grey literature. The fact that we only included qualitative studies is both a limitation and a strength. Qualitative studies add caregivers' own experiences of the usefulness of the COSP intervention; however, we consider 172 informants and 16 studies to be relatively small data sets, and generalization is not possible.

The initial idea was to exclude studies where parents received COSP through the specialist health services. However, it was considered that this might leave out important information. Therefore, all qualitative studies of COSP were included. As an extension of this, a new opportunity emerged to look more closely at differences between different groups of parents, which revealed interesting findings.

## Conclusion

The current scoping review of parents’ experiences of participating in the Circle of Security Parenting (COSP) intervention showed that parents expressed positive impacts on parents’ self-awareness, security in the parental role, emotion regulation, and parent‒child relationships and positive outcomes for children’s psychological well-being and social interaction. Parents experienced the intervention was useful to their everyday lives. The intervention was both valued and perceived as problematic for parents of children with neurodevelopmental disorders. The intervention was recommended to other parents by all target groups. The strict methodological guidelines followed make the study robust, while the small sample size and the qualitative nature of the data restrict the generalizability of the results. This synthesis contributes to filling the gaps in the knowledge of parents’ experiences and outlines practical implications for delivery and refinement. However, further research is needed using larger, quantitative, and longitudinal studies to strengthen evidence on the outcomes and effectiveness of COSP.

## Supplementary Material

supplementary.docx supplementary.docx

## Data Availability

The data that support the findings of this study are available from the corresponding author upon reasonable request.
